# Blood–brain barrier dysfunction and folate and vitamin B12 levels in first-episode schizophrenia-spectrum psychosis: a retrospective chart review

**DOI:** 10.1007/s00406-023-01572-3

**Published:** 2023-03-04

**Authors:** Mattia Campana, Lisa Löhrs, Johanna Strauß, Susanne Münz, Tatiana Oviedo-Salcedo, Piyumi Fernando, Isabel Maurus, Florian Raabe, Joanna Moussiopoulou, Peter Eichhorn, Peter Falkai, Alkomiet Hasan, Elias Wagner

**Affiliations:** 1 Department of Psychiatry and Psychotherapy, University Hospital, LMU Munich, Nussbaumstraße 7, 80336 Munich, Germany; 2 Department of Psychiatry, Psychotherapy and Psychosomatics of the University Augsburg, Medical Faculty, University of Augsburg, Augsburg, Germany; 3 Institute of Laboratory Medicine, University Hospital, LMU Munich, Munich, Germany

**Keywords:** First-episode psychosis, Schizophrenia, Cerebrospinal fluid, Folate, Vitamin B12, Blood–brain barrier

## Abstract

Vitamin deficiency syndromes and blood–brain barrier (BBB) dysfunction are frequent phenomena in psychiatric conditions. We analysed the largest available first-episode schizophrenia-spectrum psychosis (FEP) cohort to date regarding routine cerebrospinal fluid (CSF) and blood parameters to investigate the association between vitamin deficiencies (vitamin B12 and folate) and BBB impairments in FEP. We report a retrospective analysis of clinical data from all inpatients that were admitted to our tertiary care hospital with an ICD-10 diagnosis of a first-episode F2x (schizophrenia-spectrum) between January 1, 2008 and August 1, 2018 and underwent a lumbar puncture, blood-based vitamin status diagnostics and neuroimaging within the clinical routine. 222 FEP patients were included in our analyses. We report an increased CSF/serum albumin quotient (Qalb) as a sign of BBB dysfunction in 17.1% (38/222) of patients. White matter lesions (WML) were present in 29.3% of patients (62/212). 17.6% of patients (39/222) showed either decreased vitamin B12 levels or decreased folate levels. No statistically significant association was found between vitamin deficiencies and altered Qalb. This retrospective analysis contributes to the discussion on the impact of vitamin deficiency syndromes in FEP. Although decreased vitamin B12 or folate levels were found in approximately 17% of our cohort, we found no evidence for significant associations between BBB dysfunction and vitamin deficiencies. To strengthen the evidence regarding the clinical implications of vitamin deficiencies in FEP, prospective studies with standardized measurements of vitamin levels together with follow-up measurements and assessment of symptom severity in addition to CSF diagnostics are needed.

**Supplementary Information:**

The online version contains supplementary material available at 10.1007/s00406-023-01572-3.

## Background

Schizophrenia represents a broad clinical entity that is currently operationalized by symptoms and patterns of course. An increasing body of scientific evidence suggests that routine cerebrospinal fluid (CSF) analyses in patients with psychosis not only serve to detect a possible diagnosis of autoimmune encephalitis, but could also reveal underlying inflammatory or infectious processes [1]. Accordingly, a recommendation for a lumbar puncture in first-episode schizophrenia-spectrum psychosis (FEP) patients when an underlying somatic condition is suspected, is being implemented by national schizophrenia guidelines [2]. A more thorough CSF-based screening including neuronal autoantibodies of all patients with FEP has been suggested in light of recent findings [3]. Neurodegenerative processes or processes of failed neuronal regeneration [4] develop at least partially as a consequence of inflammatory responses [5]. In this specific context, a recent meta-analysis reported a higher rate of blood–brain barrier (BBB) disruption in schizophrenia than in healthy controls [6]. However, the role of BBB dysfunctions in schizophrenia’s aetiological process remains elusive [7]. Nonetheless, given the association between BBB dysfunctions and pathomechanisms implicated in the development of schizophrenia, such as disrupted glutamatergic signalling or inflammation, it can be speculated that BBB integrity, or lack thereof, might play an important role in the pathogenesis of schizophrenia and potentially psychotic disorders in general. [7, 8]. CSF is a secretion that may contain key information regarding inflammatory processes of the brain and BBB integrity. The CSF/serum albumin quotient (Qalb) is usually used as an indicator of BBB integrity [9], although it has limited differential power since it can be affected in both neuroinflammatory and cerebrovascular diseases. However, an increased Qalb is generally discussed to be a reliable sign of an active pathological process [10]. Increased levels of Qalb have been reported in psychotic disorders, indicating an impairment of BBB in these conditions [7]. Previous studies with a high number of individuals found an increased Qalb in about 16% of all FEP patients [11, 12]. In this context, vitamins might play a salient role since they are implicated in the biosynthesis of proteins which promote neuronal growth and repair. Deficiency of vitamin B12 leads to an inactivation of methionine synthase and mitochondrial methylmalonyl-CoA mutase and subsequently to the accumulation of homocysteine (Hcy) and methylmalonic acid (MMA) [13]. Hcy is a pro-inflammatory endogenous amino acid formed by the demethylation of nutritional methionine and is mostly re-methylated to methionine which requires folate as a methyl donor and vitamin B12 as a cofactor [13]. Hcy is an agonist of the neuronal N-methyl-D-aspartate receptor (NMDAr) [14]. It has been suggested that an NMDAr-dependent mechanism is involved in reducing the expression of the tight junction protein claudin-5 in brain microvascular endothelial cells, consequently promoting BBB disruption [15]. In summary, decreased levels of both vitamin B12 and folate increase levels of Hcy, which in turn could promote BBB alterations. From a pathophysiological perspective, there is evidence that certain vitamin and mineral deficiencies might be associated with an increased risk of developing schizophrenia [16–19], which might in part be related to compromise BBB integrity. Notably, the Prenatal Determinants of Schizophrenia (PDS) study analysed banked sera from a US cohort of mothers during pregnancy and could demonstrate that an elevated third-trimester Hcy level (which is inversely related to folate) was associated with a twofold increase in schizophrenia risk in offspring [20]. Moreover, serum indicators of reduced vitamin D and B in patients with schizophrenia have been found to hold significant associations with illness severity and in particular with regard to negative symptoms [17, 21]. Furthermore, these vitamin deficiencies relate to neuropsychological abnormalities observed in schizophrenia, such as hippocampal volume loss and cognitive impairments [21, 22]. The association between vitamin deficiencies (folate, B12) and the integrity of the BBB in schizophrenia remains to be investigated, especially in the light of a small study (*n* = 30) among patients with mild cognitive impairment and hyperhomocysteinemia, in which a vitamin B12-B6-folate combination appeared to improve BBB function [23]. In line with this finding, a recent randomized clinical trial among FEP patients (*n* = 120), showed that a 12-week B-vitamin supplementation may protect from cognitive decline especially in the attention and vigilance domains, particularly in patients with elevated homocysteine levels, patients with affective psychosis, and female patients [24]. Taken together, these findings show that a personalized medicine approach that considers vitamin supplementation in FEP might be beneficial [24]. With regard to patients with chronic schizophrenia, folate plus vitamin B12 supplementation improved negative symptoms in a randomized clinical trial (*n* = 140), even though treatment response was influenced by genetic variation in folate absorption [25]. In a recent meta-analysis, pooled effects showed that vitamin B supplementation (including B6, B8 and B12) reduced psychiatric symptoms significantly more than control conditions [26]. In summary, BBB abnormalities are present in at least a subgroup of FEP patients and there is evidence that vitamin deficiencies could result in BBB disruption. Therefore, we aimed to investigate the association between BBB alterations, using Qalb as a proxy, and serum vitamin B12 and folate deficiencies in a cohort of FEP patients.

## Methods

### Data extraction

In this retrospective study, we extracted data collected between 01/01/2008 and 01/08/2018 from our digital and physical medical records at the Department of Psychiatry and Psychotherapy (LMU University Hospital, Munich, Germany). Records were individually screened and data were manually extracted by two authors independently (MC, EW) using the following inclusion criteria:

- Inpatients
- At least one admission in our tertiary care hospital
- ICD-10 diagnosis of a first-episode F2x (schizophrenia-spectrum). In this context, patients admitted with a diagnosis of drug-induced FEP, which subsequently received a schizophrenia-spectrum diagnosis within the above mentioned time period for the analyses, were also included
- Available data for CSF/serum albumin quotients and at least one assessment of vitamin levels (folate or vitamin B12) within 60 days around the time point of the lumbar puncture

Medical records of patients suggesting clinical signs of a possible underlying autoimmune encephalitis (based on careful screening of all available data on clinical and physical examinations) were excluded from our analysis. This retrospective study was approved and informed consent was waived by the Ethics committee of the Faculty of Medicine (Ludwig-Maximilian University, Munich, Germany, registration number: 18–570). The study was performed in accordance with all applicable regulatory and ethical requirements.

### CSF and blood-based vitamin levels

Included patients received CSF and blood diagnostics within the clinical routine in the Department of Psychiatry and Psychotherapy. All CSF and blood analyses were conducted at the Institute of Laboratory Medicine (University Hospital, LMU Munich, Munich, Germany). Laboratory analyses comprised CSF cell counts, albumin levels in both CSF and serum and their ratio (Qalb), IgG levels in CSF and serum and their ratio, total protein in CSF and oligoclonal bands (OCBs) in both CSF and serum. Qalb values were classified as altered when outside the reference values. Reference values were adjusted by age with the following formula: Qalb = (4 + age/15) × 10^–3^, age indicated as years [27]**.** In accordance with other publications [12, 28], OCBs were classified in five pattern types as recommended by previous reports [29, 30]. The five patterns are defined as follows: pattern 1: normal CSF, pattern 2: multiple CSF restricted OCBs, pattern 3: identical OCBs in CSF and serum plus CSF restricted OCBs, pattern 4: identical OCBs in both CSF and serum, pattern 5: monoclonal bands in CSF and serum. An intrathecal synthesis of IgG is characterized by OCB patterns 2 or 3. Folate and vitamin B12 were measured according to the manufacturer’s instructions in serum/heparinized plasma samples using electrochemiluminiscent competitive binding assays on a Roche Elecsys 411e analyzer and a Roche Cobas e602 module (Roche Diagnostics GmbH, D-68305 Mannheim, Germany), respectively. The Roche Elecsys Folate and Elecsys vitamin B12 assays make use of a ruthenium labelled folate/intrinsic factor binding protein and biotin labelled folate/vitamin B12 as tracer**.** Vitamin B12 and folate levels were rated as decreased or not decreased according to cut-offs used by the Institute of Laboratory Medicine (Folate cut-off ≥ 4.6 ng/ml, vitamin B12 cut-off ≥ 250 pg/ml). Vitamin B12 cut-off was not adjusted for age, reference values for folate levels were age-adjusted (see Table 1). Over the selected time period for the analyses reported in this manuscript (01/01/2008 and 01/08/2018), the Institute of Laboratory Medicine used different folate and vitamin B12 reference values. In a pragmatic approach, we applied a single reference cut-off for vitamin B12 (250 pg/ml) as well as for folate (4.6 ng/ml) which are both broadly applied in the literature [31, 32]. In 11 cases, absolute values for vitamin B12 and folate were not specified since minimal and maximal cut-offs were exceeded. In order to provide an estimate of these absolute vitamin B12 and folate values in our cohort, we considered values smaller than the lower cut-offs (*n* = 2) as being equal in value to the lower cut-off (vitamin B12 < 150 pg/ml and folate < 2 ng/ml) and, similarly, values greater than the upper reference (*n* = 9) as being equal in value to the upper reference (folate > 20 ng/ml or folate > 24 ng/ml).

**Table 1:** Demographic and clinical characterization of the included patients

^Demographic and clinical information (*n* = patients included in analyses)^	^Complete cohort (*n* = 222)^	^Vitamin B12 OR Folate decreased (*n *= 39)^	^Vitamin B12 AND Folate within range (*n *= 183)^	^Vitamin deficiencies vs vitamins within range^		
	Mean ± SD	Mean ± SD (*n*)	Mean ± SD (*n*)	*Z*	*df*	*p*
Mean age at time of lumbar puncture (years) (*n* = 222)	34.89 ± 15.77	35.90 ± 17.65 (*n* = 39)	34.67 ± 15.38 (*n* = 183)	−0.19	1	0.851^a^
Mean duration between first symptoms and lumbar puncture (months) (*n* = 140)	13.37 ± 25.35	21.70 ± 47.74 (*n* = 27)	11.38 ± 15.74 (*n* = 113)	−0.73	1	0.466^a^

	% (absolute)	% (absolute)	% (absolute)	*χ*^2^	*df*	*p*
Gender (female/male) (*n* = 222)	46.40/53.60 (103/119)	43.59/56.41 (17/22)	46.99/53.01 (86/97)	0.15	1	0.699
Diagnosis^*^ (*n* = 222)						
Schizophrenia (ICD-10: F20)	46.40 (103)	41.03 (16)	47.54 (87)	6.34	5	0.275
Schizotypal disorder (ICD-10: F21)	3.15 (7)	7.69 (3)	2.19 (4)			
Persistent delusional disorder (ICD-10: F22)	8.11 (18)	5.13 (2)	8.74 (16)			
Acute and transient psychotic disorder (ICD-10: F23)	22.97 (51)	17.95 (7)	24.04 (44)			
Schizoaffective disorder (ICD-10: F25)	9.01 (20)	12.82 (5)	8.20 (15)			
Others	10.36 (23)	15.38 (6)	9.29 (17)			
Antipsychotic treatment (*n* = 210)	96.67 (203)	100 (37)	95.95 (166)		1	0.252^b^
Antipsychotic treatment with 1 AP (*n* = 210)	69.05 (145)	75.68 (28)	67.63 (117)	0.92	1	0.337
Antipsychotic treatment with 2 or more APs (*n* = 210)	27.62 (58)	24.32 (9)	28.33 (49)	0.24	1	0.621
Positive family history for psychiatric illnesses (*n* = 204)	51.47 (105)	42.86 (15)	53.25 (90)	1.25	1	0.263
Comorbidities						
Neurological comorbidity (*n* = 222)	9.91 (22)	15.38 (6)	8.74 (16)	1.59	1	0.208
Diabetes type I or II (*n* = 222)	4.50 (10)	5.13 (2)	4.37 (8)		1	0.689^b^
Cardiovascular disease (*n* = 220)	15.45 (34)	15.38 (6)	15.47 (28)	< 0.01	1	0.989
Cancer (*n* = 219)	8.22 (18)	10.53 (4)	7.73 (14)		1	0.525^b^
Lung disease (*n* = 221)	8.14 (18)	10.26 (4)	7.69 (14)		1	0.532^b^
Active smoker (*n* = 215)	34.42 (74)	43.24 (16)	32.58 (58)	1.54	1	0.214
Active drug abu*s*e (at least 1 psychotropic substance)** (*n* = 219)	29.22 (64)	25.64 (10)	30 (54)	0.29	1	0.587
Cannabis abuse (*n* = 222)	16.67 (37)	17.95 (7)	16.39 (30)	0.06	1	0.813

Vitamin serum levels	Mean ± SD	Mean ± SD (*n*)	Mean ± SD (*n*)
Vitamin B12 level pg/ml (*n* = 222)	481.98 ± 261.87	203.65 ± 36.62 (*n* = 23)	514.15 ± 257.60 (*n* = 199)
Folate level ng/ml (*n* = 217)	9.46 ± 4.74	7.12 ± 4.71 (*n* = 22)	9.73 ± 4.68 (*n* = 195)

	% (absolute)
Vitamin B12 decreased**** (*n* = 222)	10.36 (23)
Folate decreased*** (*n* = 217)	11.06 (24)
Vitamin B12 OR Folate decreased**** (*n* = 222)	17.57 (39)

Cerebrospinal fluid assessment	Mean ± SD	Mean ± SD (*n*)	Mean ± SD (*n*)	*Z*	*df*	*p*
Albumin level, g/l (*n* = 222)	0.25 ± 0.11	0.22 ± 0.08 (*n* = 39)	0.25 ± 0.12 (*n* = 183)	−0.89	1	0.373^a^
Qalb (CSF/serum) (*n* = 222)	5.47 ± 2.40	5.08 ± 1.74 (*n* = 39)	5.56 ± 2.51 (*n* = 183)	−0.71	1	0.479^a^
IgG, g/l (*n* = 221)	0.03 ± 0.02	0.03 ± 0.03 (*n* = 38)	0.03 ± 0.02 (*n* = 183)	−1.30	1	0.194^a^
IgG ratio (CSF/serum) (*n* = 221)	2.79 ± 2.06	3.14 ± 4.23 (*n* = 38)	2.71 ± 1.21 (*n* = 183)	−0.43	1	0.670^a^
Total protein level, g/l (*n* = 222)	0.38 ± 0.27	0.44 ± 0.57 (*n* = 39)	0.37 ± 0.14 (*n* = 183)	−0.27	1	0.785^a^
Number of cells, /µL (*n* = 222)	1.58 ± 1.91	1.31 ± 1.47 (*n* = 39)	1.63 ± 2 (*n* = 183)	−1.23	1	0.220^a^

	% (absolute)	% (absolute)	% (absolute)	*χ*^2^	*df*	*p*
Altered Qalb*** (*n* = 222)	17.12 (38)	7.69 (3)	19.13 (35)		1	0.103^b^
Oligoclonal bands (*n* = 171)						
No oligoclonal bands, type 1	72.51 (124)	72.73 (24)	72.46 (100)	0.08	3	0.994
Oligoclonal bands type 2	2.92 (5)	3.03 (1)	2.90 (4)			
Oligoclonal bands type 3	8.19 (14)	9.09 (3)	7.97 (11)			
Oligoclonal bands type 4	16.37 (28)	15.15 (5)	16.67 (23)			
Oligoclonal bands type 5	0 (0)					

Brain MRI	% (absolute)	% (absolute)	% (absolute)	*χ*^2^	*df*	*p*
Any brain MRI pathologies (*n* = 212)	40.09 (85)	44.74 (17)	39.08 (68)	.42	1	0.519
WML(s) (*n* = 212)	29.25 (62)	36.80 (14)	27.60 (48)	1.29	1	0.256
Lesion(s) suggestive of MS (*n* = 212)	1.89 (4)	0 (0)	2.3 (4)		1	1^b^

*AP* antipsychotic, *CSF* cerebrospinal fluid, *Qalb* cerebrospinal fluid/serum albumin quotient, *MRI* magnetic resonance imaging, *MS* multiple sclerosis, *WML* White matter lesion, *χ*^2^ chi-square, *Z* Z statistic, *df* degrees of freedom, *p* p-value, *SD* standard deviation

^a^Comparison by Mann–Whitney *U*-test

^b^Comparison by two-sided Fisher’s exact test

*Diagnosis according to International Statistical Classification of Diseases and Related Health Problems criteria, version 10 (ICD-10)

**Illegal drug abuse, cannabis abuse and alcohol abuse included

***Adjusted for age Qalb age-dependent reference value = (4 + age/15) × 10^–3^, age indicated as years; Folate = cut-off 4.6 ng/ml. The aforementioned folate cut-off was used for patients >  = 18 years old, no reference values were used for patients younger than 18 years old

****Vitamin B12 cut-off 250 pg/ml. Cut-off points for Vitamin B12 were not adjusted to age

### Brain magnetic resonance imaging (MRI)

As part of the diagnostic clinical routine, a brain MRI is usually offered to every FEP patient admitted in our Department of Psychiatry and Psychotherapy. We examined the neuroradiological evaluation reported in the patient medical files and recorded brain MRI pathologies if they included any alterations uncommon for the patient’s age group. Recorded brain MRI pathologies included non-specific WML and lesions suggestive of multiple sclerosis (MS). Every routine MRI protocol comprised T1-weighted and T2-weighted sequences (1.5 and 3 Tesla scanners). Furthermore, additional DWI (axial) and FLAIR (coronal) sequences were performed if indicated by an experienced neuroradiologist.

### Groups comparison

Patients included in our analyses were divided in two sub-groups based on vitamin status. Both demographic and clinical variables were compared between patients with vitamin B12 OR folate serum levels under the cut-off values and patients with vitamin B12 AND folate serum levels within reference range.

### Statistical analyses

Analyses were executed using IBM SPSS version 28.0 for Windows. The significance level was set at *α* = 0.05. Descriptive statistics included frequencies, sum, mean and standard deviation (SD). All these parameters were applied for continuous variables and frequencies were applied to present dichotomous variables. Chi-square tests were applied to compare differences in categorical variables between groups (two-sided Fisher’s exact test was applied for cell count < 5 for 2 × 2 tables). Cramer’s *V* tests were adopted to test for possible associations between categorical variables. Continuous variables were tested for normality with Kolmogorov–Smirnov tests, a violation of the normal distribution was defined as *p* < 0.05. Mann–Whitney-*U* tests were used to compare differences between continuous non-parametric variables. The following results must be considered exploratory since they include multiple different statistical analyses that were not corrected for multiple testing.

## Results

### Characterization of our cohort

A total of *n* = 222 FEP patients were included in our cohort. Mean age was 34.9 years (SD = 15.8), with 53.6% (*n* = 119) male and 46.4% (*n* = 103) female patients. All of the included patients received a diagnosis in the schizophrenia-spectrum (F2x according to ICD-10), whereby almost half of the sample (46.4%, *n* = 103) received a diagnosis of schizophrenia (F20 according to ICD-10). The mean duration of illness between onset of first symptoms and lumbar puncture was 13.4 months (SD = 25.4, *n* = 140). 69.1% (*n* = 145) of the patients were treated with one (first- or second generation) antipsychotic and 27.6% (*n* = 58) received two or more antipsychotics. Half of the cohort (51.5%, *n* = 105) had a first and/or second-degree relative with a psychiatric condition. 34.4% (n = 74) of the patients were active smokers while 29.2% (*n* = 64) of the patients showed active drug abuse of at least one substance other than tobacco. Among them, 16.7% (*n* = 37) consumed cannabis anywhere from sporadically to daily. A small number of patients (9.9%, *n* = 22) were diagnosed with concurrent neurological disorders, while in 3.6% of cases (*n* = 8) a gastrointestinal disorder was reported (for further details please refer to Table S1). Less than 5% of patients (4.5%, *n* = 10) were diagnosed with diabetes type I or II. 15.5% (*n* = 34) presented a diagnosis of a concomitant cardiovascular condition. Finally, 8.2% (*n* = 18) of the patients were diagnosed with cancer (active or in remission) and 8.1% (*n* = 18) were diagnosed with a lung disease (see Table 1).

### Vitamin B12, folate and CSF parameters

Among the *n* = 222 patients who received a vitamin B12 assessment, the mean vitamin B12 serum level was 481.98 pg/ml (SD = 261.9). A total of *n* = 217 patients received a folate assessment with mean folate serum levels of 9.46 ng/ml (SD = 4.7). *n* = 23 (10.4%) patients showed decreased vitamin B12 serum levels. Similarly, *n* = 24 (11.1%) patients presented decreased folate serum levels. For patients younger than 18 years (*n* = 5), no folate reference values were used by the laboratory. Nevertheless, their mean folate value was 8.78 ng/ml (SD = 5.0) and thus above the adult cut-off value (4.6 ng/ml). Mean albumin CSF level was 0.25 g/l (SD = 0.1) with a mean Qalb of 5.47 (SD = 2.4). 17.1% (*n* = 38) of the 222 patients showed an altered age-adjusted Qalb. Mean IgG CSF levels were 0.03 g/l (SD = 0.02) with a mean IgG CSF/serum ratio of 2.79 (SD = 2.1). Total CSF protein level was 0.38 g/l (SD = 0.27) whereas the mean number of cells in CSF was 1.58/µL (SD = 1.9). Among patients tested for OCBs both in CSF and serum (*n* = 171), n = 124 patients (72.5%) showed no OCBs (pattern 1), *n* = 5 patients (2.9%) showed OCBs pattern 2, OCBs pattern 3 were present in *n* = 14 patients (8.2%) while OCBs pattern 4 were reported in *n* = 28 patients (16.4%). No OCBs pattern 5 were found in our cohort (see Table 1).

### Brain MRI

Brain MRI data were available for a total of *n* = 212 patients. Any brain MRI abnormal findings were detected in *n* = 85 (40.1%) patients. WMLs were present in *n* = 62 (29.3%) cases, with 1.9% (*n* = 4) cases having lesions suggestive of MS (see Table 1).

### Comparison of demographical and clinical variables according to vitamin status

A detailed comparison of demographical and clinical variables between patients with vitamin B12 or folate serum levels under the cut-off values and patients with vitamin B12 and folate serum levels within reference values can be found in Table 1. Overall, no statistically significant differences were found when comparing the two groups. Of note, there was no significant difference when comparing altered Qalb between groups (df = 1, Cramer’s *V* = 0.12, *p* = 0.103). Similarly, no significant difference was found when comparing for WMLs rate between groups ($\chi_{(1)}^{2}$ = 1.29, Cramer’s *V* = 0.08, *p* = 0.256) (see Table 1).

## Discussion

To our knowledge, this is the largest FEP cohort with a schizophrenia-spectrum disorder having received CSF diagnostics and vitamin B12 and folate serum assessments. The aim of this investigation was to explore the relationship between BBB dysfunction represented by altered age-adjusted Qalb and vitamin B12 and folate serum levels in FEP patients. The cohort presented here was part of a larger clinical population, that was previously described by our research group [12]. Corresponding to the aforementioned cohort, we report signs of BBB dysfunction (increased Qalb) in 17.1% of the included population. Moreover, a relatively small percentage of the cohort showed decreased vitamin B12 or folate serum levels (10.4% and 11.1% respectively). Put in perspective with data from healthy controls of similar age [33], only 1.0% of omnivores (5.7% of vegetarians and 7.5% of vegans) presented decreased vitamin B12 levels. Nevertheless, in the healthy control cohort a lower vitamin B12 cut-off was applied (150 pg/ml) than in our FEP analyses (250 pg/ml), which could explain the large difference in the prevalence of vitamin B12 deficiency. In the aforementioned study among healthy subjects [33], decreased folate serum levels were reported in 58% of omnivores (30.2% of vegetarians and 13.2% of vegans), a greatly higher level than the 11.1% reported in our cohort, although the cut-off used in the aforementioned publication was set at 6.6 ng/ml and thus higher than the one used in our approach (4.6 ng/ml). As mentioned before, definitions for vitamin B12 and folate deficiency are highly heterogeneous and might explain the heterogeneity in the prevalence of hypovitaminoses across the literature. In this context, it must be noted that confounding factors such as nutritional and lifestyle habits or body-mass-index were not recorded, so that it remains difficult to pinpoint a cause for the higher prevalence of vitamin B12 deficiency and the reduced prevalence of decreased folate levels in our cohort compared to the healthy control cohort. When assessing the relation between BBB dysfunction and vitamin B12 or folate deficiency, no statistically significant association could be found. Nevertheless, a potential causal relationship between BBB disruption and vitamin deficiencies cannot be excluded based on our cross-sectional retrospective data.

In our cohort, the mean duration of illness was 13.4 months, which could imply that not all patients with a vitamin B12 deficiency already had low vitamin B12 serum levels at an early disease stage. It is known that a vitamin B12 deficiency can be preclinical for more than 3–5 years [34], thus the results have to be interpreted with caution. Further investigation is needed to evaluate the course of especially FEP patients and vitamin B12 levels, starting with the prodromal stage of psychosis and following the course of the disease.

When assessing brain MRI data, we found alterations in 40.1% of patients. A much lower rate than the approximately 70% reported in another large cohort which was not restricted to FEP patients [11]. In our cohort we report WMLs in 29.3% of brain MRI scans. As a reference for comparison: WMLs occurred in around 5.3% of healthy controls with a similar mean age [35]. We found no statistically significant association between WMLs occurrence and increased Qalb levels as the pathogenesis of WMLs remains controversial and in need of more appropriate investigations [36].

A major limitation of the present study is its retrospective design. As a consequence, several patients did not receive all assessments included in this analysis. Thus, from a cohort of initially 687 FEP patients, only 222 underwent a lumbar puncture as part of the clinical routine and received an assessment of vitamin B12 and folate levels within 60 days prior or following a lumbar puncture. Hypotheses for the lack of CSF diagnostics in a substantial part of the cohort are difficult to test retrospectively, whereas vitamin B12 and folate deficiencies are rarely examined in general and rather have the status of optional examinations. This is astonishing, since there is evidence that supplementation of certain vitamins may reduce psychiatric symptoms in people with schizophrenia [26]. Of note, the assessment of vitamin B12 and folate serum levels among FEP patients is currently suggested as facultative in some guidelines [37] or completely omitted in others [38]. Moreover, in a few cases absolute values for vitamin B12 and folate were missing in our analyses, since these values were not specified when minimal and maximal cut-offs were exceeded. Since different cut-offs for vitamin B12 and folate levels were used in our tertiary care hospital during the period of interest, we used a pragmatic approach for our analyses. Of note, applying different reference values with lower cut-offs would not have a substantial impact on the results of our comparison analyses. Furthermore, a vitamin B12 deficiency syndrome can be associated with normal vitamin B12 serum levels. Around 80% of the circulating biomarker vitamin B12, determined in the serum is protein-bound to haptocorrin and therefore not bioavailable for cellular uptake [39]. In addition, the serum and cellular levels of vitamin B12 differ, so that the diagnostic value of a serum sample is reduced [39]. Nevertheless, serum B12 remains a valuable biomarker to better characterize the prognosis and status of several diseases [40], but it has a limited diagnostic value as a stand-alone biomarker. Complementing the diagnostics of a vitamin B12 deficiency through assessment of serum holotranscobalamin and MMA can increase the sensitivity in assessing a sub-clinical functional vitamin B12 deficiency [41] and should be considered in future studies. This could be particularly important for FEP cohorts, since a functional B12 deficiency may be present even if it cannot be detected by measuring vitamin B12 serum levels alone. Given the association between reduced vitamin D and illness severity [21], a more thorough assessment of the vitamin status might be recommended for future clinical practice. However, among the selected population only data regarding vitamin B12 and folate were available. For this reason, determining the status of other key vitamins was not possible. Given the retrospective design of this study, it was not possible to determine whether patients presenting a B12 or folate deficiency received any vitamin supplementation. Although lumbar punctures were performed within days of the vitamin assessment, a possible role of supplementation in offsetting a potential BBB dysfunction cannot be ruled out. It is important to mention the relative inhomogeneity regarding characteristics, e.g. age and comorbidities in our cohort. This is due to the retrospective study design with a large sample size, hence a broader and less fine selected patient’s population is assessed which ultimately better represents real world settings. Finally, no information were available regarding symptom severity as measured by standardized questionnaires, limiting the investigation of possible correlations between biological markers and impairments in different symptom domains.

In summary, our study contributes to an increased knowledge about vitamin deficiencies in FEP patients. To draw potential associations between vitamin deficiencies and symptoms severity, prospective studies are needed. We recommend that these studies should employ CSF diagnostics beyond the clinical routine, standardized (follow-up) measurements of vitamin levels as well as assessments of cognitive functioning and symptom severity to further foster the evidence with regard to the prevalence and clinical implications of vitamin deficiency syndromes in FEP. Overall, this study provides epidemiological evidence regarding vitamin deficiencies and BBB dysfunctions in first-episode schizophrenia-spectrum psychosis. We believe this work could raise awareness on this important topic and pave the way for prospective implementation of vitamin assessments in the clinical practice and especially in the diagnostic algorithms of psychosis.

### Supplementary Information

Below is the link to the electronic supplementary material.

Supplementary file1 (DOCX 14 KB)

## Data Availability

The datasets used and/or analysed during the current study are available from the corresponding author on reasonable request.

## Abbreviations

BBB: Blood-brain barrier
CSF: Cerebrospinal fluid
FEP: First-episode schizophrenia-spectrum psychosis
Hcy: Homocysteine
MMA: Methylmalonic acid
MRI: Brain magnetic resonance imaging
MS: Multiple sclerosis
NMDAr: N-methyl-D-aspartate receptor
OCBs: Oligoclonal bands
PDS: Prenatal determinants of schizophrenia
Qalb: Cerebrospinal fluid /serum albumin quotient
WML: White matter lesions
