# Supplementary material for: Blood–brain barrier dysfunction and folate and vitamin B12 levels in first-episode schizophrenia-spectrum psychosis: a retrospective chart review
Source: Eur Arch Psychiatry Clin Neurosci. 2023 Mar 4;273(8):1693–701. doi: 10.1007/s00406-023-01572-3 (PMC10713685; doi:10.1007/s00406-023-01572-3)
Supplement: Supplementary file 1 — Supplementary file1 (DOCX 14 KB) [file 406_2023_1572_MOESM1_ESM.docx]

**Table S1: List of neurological comorbidities**

| **Case number** | **Neurological condition** |
| --- | --- |
| Case n. 1 | Traumatic brain injury during childhood with epileptic seizures. From age 17 onwards no more seizures even though no anticonvulsive medication was taken. |
| Case n. 2 | Developmental venous anomaly in the right temporal lobe. |
| Case n. 3 | Meningoencephalitis during childhood (age of 11 years). |
| Case n. 4 | Traumatic brain injury during childhood (age of 4 years) with ongoing reduced motor and sensory functions in the right extremities. |
| Case n. 5 | Traumatic brain injury in 2003 with subsequent neuropsychological deficits. |
| Case n. 6 | Stroke in 1986 (left middle cerebral artery). |
| Case n. 7 | Right frontal lobe hypodensity following traumatic brain injury during childhood (at age of 5 years). |
| Case n. 8 | Diabetic neuropathy. |
| Case n. 9 | Neuroborreliosis with ipsilateral facial palsy. |
| Case n. 10 | Left hemispheric ischemia with subsequent aphasia and facial palsy. |
| Case n. 11 | Spinal disc herniation with right foot extensor palsy. |
| Case n. 12 | Meningitis during childhood (at age of 11 years) with epileptic seizures. Seizure-free after successful treatment of meningitis. |
| Case n. 13 | Traumatic brain injury one year before lumbar puncture. |
| Case n. 14 | Uraemic neuropathy, recurrent laryngeal nerve paralysis. |
| Case n. 15 | Epilepsy from age 2 onwards. Seizure-free since age 14. |
| Case n. 16 | Suspected multiple sclerosis. |
| Case n. 17 | Vitamin B12 deficiency neuropathy. |
| Case n. 18 | Idiopathic peripheral neuropathy. |
| Case n. 19 | Charcot–Marie–Tooth disease (CMT1A). |
| Case n. 20 | Idiopathic intracranial hypertension. |
| Case n. 21 | Cerebral palsy. |
| Case n. 22 | Migraine. |

**List of gastroenterological comorbidities**

| **Case number** | **Gastroenterological condition** |
| --- | --- |
| Case n. 1 | Chronic gastritis. |
| Case n. 2 | Chronic gastritis. |
| Case n. 3 | Suspected chronic gastritis. |
| Case n. 4 | Suspected chronic gastritis. |
| Case n. 5 | Chron´s disease. |
| Case n. 6 | Gastroesophageal reflux disease. |
| Case n. 7 | Gastroesophageal reflux disease. |
| Case n. 8 | Irritable bowel syndrome. |
